# The fungi effector VmRnt2 from *Valsa mali* modulates host transcription factor to suppress immunity in apple

**DOI:** 10.1093/hr/uhag054

**Published:** 2026-02-26

**Authors:** Hailong Liu, Pujiang Deng, Xing Gao, Shasha Chen, Qiyue Zhang, Liangsheng Xu, Lili Huang

**Affiliations:** State Key Laboratory for Crop Stress Resistance and High-Efficiency Production, College of Plant Protection, Northwest A&F University, Yangling, Shaanxi, China; State Key Laboratory for Crop Stress Resistance and High-Efficiency Production, College of Plant Protection, Northwest A&F University, Yangling, Shaanxi, China; State Key Laboratory for Crop Stress Resistance and High-Efficiency Production, College of Plant Protection, Northwest A&F University, Yangling, Shaanxi, China; State Key Laboratory for Crop Stress Resistance and High-Efficiency Production, College of Plant Protection, Northwest A&F University, Yangling, Shaanxi, China; State Key Laboratory for Crop Stress Resistance and High-Efficiency Production, College of Plant Protection, Northwest A&F University, Yangling, Shaanxi, China; State Key Laboratory for Crop Stress Resistance and High-Efficiency Production, College of Plant Protection, Northwest A&F University, Yangling, Shaanxi, China; Northwest A&F University ShenZhen Research Institute, Shenzhen, Guangdong 518000, China; State Key Laboratory for Crop Stress Resistance and High-Efficiency Production, College of Plant Protection, Northwest A&F University, Yangling, Shaanxi, China

## Abstract

Apple Valsa canker (AVC), a disease instigated by *Valsa mali* (syn. *Cytospora mali*), poses a significant global threat to apple cultivation. Throughout its infection process, *V. mali* introduces an array of effector proteins into the host cells aimed at undermining the host immune defenses. The exact molecular mechanisms through which these effectors manipulate host transcription factors (TFs) to promote pathogenesis are not fully understood. This study identifies a ribonuclease T2-like effector, VmRnt2, that notably inhibits INF1-triggered cell death, chitin-induced reactive oxygen species bursts, and callose deposition. Knockout of the *VmRnt2* gene markedly reduced the virulence of *V. mali*, without impacting fungal growth or spore production. Conversely, heterologous expression of *VmRnt2* in *Nicotiana benthamiana* and apple markedly enhanced susceptibility to infections by *Sclerotinia sclerotiorum* and *V. mali*, respectively, highlighting its pivotal role in facilitating pathogenicity. VmRnt2 was found to interact specifically with an apple TF, MdMYB44, which belongs to the myeloblastosis (MYB) family of proteins. Further functional assays revealed that overexpression of *MdMYB44* in apple enhances resistance to *V. mali*. Additionally, MdMYB44 was shown to bind specifically to the promoter of the defense-related gene *MdPR1A*, subsequently activating its transcription. Importantly, during *V. mali* infection, VmRnt2 disrupts the DNA-binding activity of MdMYB44. Collectively, our results elucidate how *V. mali* employs VmRnt2 to compromise MdMYB44-mediated immune regulation, thereby facilitating the pathogen’s colonization of apple trees.

## Introduction

Plants employ a dual-layered innate immune system to defend themselves against pathogens: pathogen-associated molecular pattern-triggered immunity (PTI) and effector-triggered immunity (ETI), which are mediated by pattern recognition receptors (PRRs) and nucleotide-binding leucine-rich repeat (NLR) receptors respectively. To circumvent these defense mechanisms, pathogens deliver an array of effector proteins that suppress both PTI and ETI, thereby facilitating effector-triggered susceptibility [1–3]. Recent studies demonstrate that PTI and ETI can mutually reinforce each other, synergistically enhancing disease resistance [4, 5]. Despite these sophisticated defense systems, pathogens can still undermine plant immunity by targeting crucial immune components with effectors, thus enabling infection and colonization [6]. Consequently, the identification and functional characterization of pathogen effectors and their host targets are critical for understanding the molecular underpinnings of pathogen virulence.

Transcription factors (TFs) are pivotal DNA-binding proteins that orchestrate gene expression critical for plant development and defense mechanisms [7, 8]. Concurrently, during host infection, fungal pathogens deploy effector proteins that specifically target defense-related TFs to disrupt the transcriptional regulation of immunity [9, 10]. These effectors utilize a variety of molecular mechanisms to impede TF functionality. For instance, in chickpea, the *Ascochyta rabiei* effectors ArPEC25 binds to the TF CaβLIM1a, subsequently inhibiting its interaction with the *CaPAL1* promoter and reducing the biosynthesis of phenylpropanoids and lignin [11]. Similarly, in *Arabidopsis thaliana*, the *Verticillium dahliae* effectors VdSCP41 and Vd6317 compromise the transcriptional activities of the TFs SARD1 and AtNAC53, respectively, thereby facilitating infection [12, 13]. In wheat, the *Puccinia striiformis* f. sp. *tritici* (*Pst*) effector PstGSRE1 obstructs the nuclear translocation of the TF TaLOL2, thus suppressing the hypersensitive response [14]. Another *Pst* effector, Pst21674, hampers the multimerization of TaASR3, a process crucial for the activation of defense-related genes, thereby enhancing host susceptibility [15]. Additionally, the effector PevD1 prolongs the stability of the senescence-associated NAC TF ORE1 by disrupting its interaction with the ubiquitin E3 ligase NLA, which contributes to increased ethylene production and enhanced pathogen virulence [16].

The myeloblastosis (MYB) family constitutes one of the most extensive groups of plant TFs, with its members playing crucial roles in plant growth, development, and response to stress [17, 18]. Recent functional analyses have shown that several MYB TFs are directly targeted by fungal effectors to enhance pathogen virulence. For example, the *Colletotrichum gloeosporioides* effector CgNLP1 undermines plant immunity by disrupting the nuclear localization of the necrosis-induced TF HbMYB8-Like [19]. Similarly, the *Fusarium oxysporum* f. sp. *cubense* effector FSE1 interacts with the banana MYB TF MaEFM-like within the nucleus, inhibiting MaEFM-induced cell death and diminishing disease resistance [20]. Moreover, The *Pst* effector protein Pst15882 interacts with TaMYB50, mitigating its repression of the susceptibility gene *TaSWEET14d* and thereby facilitating infection [21]. Consequently, elucidating the interactions between plant TFs and pathogen effectors is pivotal for understanding the strategies pathogens employ to manipulate host immunity.

Apple Valsa canker (AVC), caused by the necrotrophic fungus *Valsa mali* (syn. *Cytospora mali*), poses a significant threat to global apple production [22, 23]. Though prior research has demonstrated that *V. mali* secretes effector proteins to undermine plant immunity [24–29], the specific mechanisms by which *V. mali* effectors target apple TFs have not been thoroughly investigated. In this study, we present evidence that the *V. mali* effector protein VmRnt2 interacts with the apple TF MdMYB44, suppressing its transcriptional activation of the defense gene *MdPR1A* and thereby facilitating fungal infection. Our findings elucidate a novel molecular mechanism through which a fungal effector targets a MYB TF to suppress plant immunity during pathogen invasion.

## Results

### *VmRnt2* is essential for full virulence of *V. mali*

**Figure 1:**
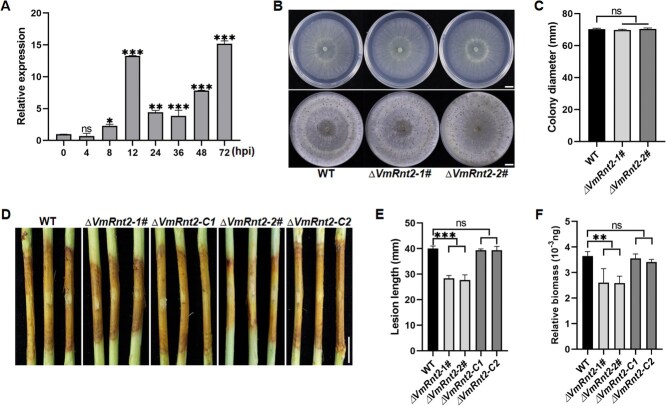
VmRnt2 is essential for the virulence of *V. mali* in apple. **(A)** Expression profile of *VmRnt2* during *V. mali* infection, as determined by quantitative reverse transcription PCR (qRT-PCR). *VmRnt2* transcript levels were measured at specified time points following inoculation of apple twigs. **(B, C)** Morphology and colony size of the wild-type (WT) strain and *VmRnt2* deletion mutants (*ΔVmRnt2–1*# and *ΔVmRnt2-2*#) grown on potato dextrose agar (PDA) plates incubated at 25°C for 48 h in darkness. Scale bar represents 1 cm. **(D-F)** Disease symptoms (D), lesion length (E), and relative fungal biomass (F) in apple twigs inoculated with the wild-type strain, *ΔVmRnt2* deletion mutants, or *ΔVmRnt2* complemented strains at 5 days post-inoculation (dpi). Scale bar represents 1 cm. The *G6PDH* gene served as the internal control. Data represent the mean ± standard deviation of three biological replicates. Asterisks indicate statistically differences (Student’s *t*-test, ^*^P < 0.05, ^**^P < 0.01, ^***^P < 0.001). Experiments were repeated three times with similar results.

In our previous research aimed at identifying effectors related to the pathogenicity of *V. mali*, we conducted a proteomic analysis on the wild-type strain 03-8 during its infection of apple twigs [30]. This analysis revealed that the secreted protein KUI72745.1, subsequently renamed VmRnt2, was significantly upregulated during the infection stage. Quantitative reverse transcription PCR (qRT-PCR) analysis confirmed that the expression of *VmRnt2* was significantly induced at 12 and 72 h postinoculation (hpi) **(**Fig. 1A**)**, underscoring its potential role in the interaction between *V. mali* and apple. VmRnt2 encodes a 420-amino acid protein with a predicted signal peptide (SP) and is functionally annotated as a ribonuclease T2-like protein **(**Supplementary Fig. S1B**)**, with a ribonuclease T2 domain located at its N-terminus **(**Supplementary Fig. S1B**)**. To further explore the role of *VmRnt2*, we generated knockout mutants (*ΔVmRnt2*) and a complementation strain (*ΔVmRnt2-C*) using polyethylene glycol (PEG)-mediated protoplast transformation, verifying the transformants by PCR **(**Supplementary Fig. S2**)**. While the *ΔVmRnt2* mutants displayed no change in mycelial growth rates or conidiation compared to the wild type (WT) **(**Fig. 1B and C**)**, pathogenicity assays on apple twigs revealed significantly reduced virulence in these mutants relative to both WT and *ΔVmRnt2-C*  **(**Fig. 1D and E**)**. There was also a significant decrease in relative fungal biomass in the mutants (Fig. 1F). These findings confirm that *VmRnt2* is crucial for the full virulence of *V. mali*.

### VmRnt2 carries a functional secretory SP

To functionally validate the predicted SP of VmRnt2, we employed a yeast signal sequence trap system. The N-terminal 20 amino acids, corresponding to the predicted SP, were cloned and fused into the vector pSUC2. The assay results demonstrated that the SP construction of VmRnt2, similar to Avr1b used as a positive control, enabled the invertase-deficient yeast strain YTK12 to grow on a raffinose-containing medium (YPRAA; Supplementary Fig. S3A). Conversely, YTK12 harboring the empty pSUC2 vector (negative control) failed to grow on YPRAA (Supplementary Fig. S3A). Additionally, a domain-swap experiment in which the SP of INF1 was replaced with that of VmRnt2 led to the chimeric protein triggering cell death in *Nicotiana benthamiana*  **(**Supplementary Fig. S3B**)**. Together, these results unequivocally establish that the predicted SP of VmRnt2 is functional and facilitates secretion.

### VmRnt2 has RNase activity

To investigate the RNase activity of VmRnt2, a GST-tagged version of the VmRnt2 protein was expressed and purified using *Escherichia coli* DE3. A multiple sequence alignment between VmRnt2 and its homologues identified two conserved histidine residues within the ribonuclease T2 domain **(**Supplementary Fig. S4**)**. Site-directed mutagenesis was employed to replace these putative catalytic histidine residues with alanine, resulting in the recombinant mutant protein VmRnt2^H80/H142A^, which was expressed under the same conditions. The RNase activities of both the recombinant VmRnt2 and the mutant VmRnt2^H80/H142A^ were assessed by incubating them with apple total RNA. While VmRnt2 effectively degraded the RNA, both VmRnt2^H80/H142A^ and GST-tagged control protein (negative control) showed no RNA degradation when tested under identical conditions **(**Supplementary Fig. S5**)**. These findings confirm that VmRnt2 exhibits RNase activity and that the catalytic residues His80 and His142 are crucial for its enzymatic function.

### VmRnt2 suppresses INF1-induced cell death in *N. benthamiana* and enhances plant susceptibility to pathogens in tobacco and apple

**Figure 2:**
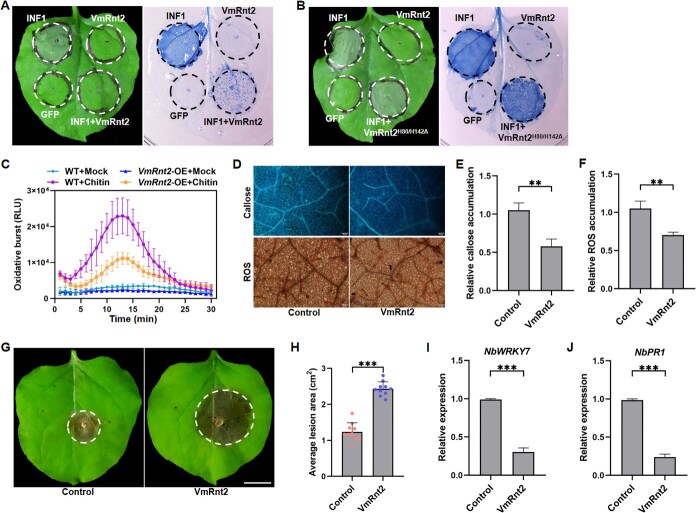
VmRnt2 inhibits the immune response of *N. benthamiana.*  **(A)** VmRnt2 suppresses INF1-induced cell death in *N. benthamiana* (left) and cell death was assessed by trypan blue staining (right). **(B)** VmRnt2^H80/H142A^ cannot suppresses INF1-induced cell death. **(C)** Overexpression of VmRnt2 in *N. benthamiana* reduces chitin-triggered ROS burst. **(D)** Deposition of callose and accumulation of ROS in *N. benthamiana* in response to VmRnt2 expression. Leaves were infiltrated with *A. tumefaciens* carrying either the empty GFP vector (Control) or the VmRnt2-GFP construct (VmRnt2) and imaged at 48 h post-infiltration. Scale bar represents 50 μm. **(E, F)** Quantification of callose deposition (E) and ROS accumulation (F) from images as in (D), measured using ImageJ (intensity per image). Quantification of callose and ROS was performed per image with ImageJ. **(G)** VmRnt2 suppresses *N. benthamiana* resistance to *S. sclerotiorum*. *N. benthamiana* leaves were infiltrated with *A. tumefaciens* carrying VmRnt2-GFP or GFP separately 48 h before pathogen inoculation. **(H)** Area of lesions on *N. benthamiana* leaves infected by *S. sclerotiorum*. Scale bar represents 1 cm. **(I, J)** Relative expression levels of defense-associated genes *NbWRKY7* and *NbPR1*. *NbActin* was used as the internal reference gene. All data represent the mean ± standard deviation of three independent replicates. Asterisks indicate significant differences (Student’s *t*-test, ^**^P < 0.01, ^***^P < 0.001). Experiments were repeated three times with similar results.

Transient overexpression in *N. benthamiana* leaves is an effective and commonly employed technique for investigating inhibition of elicitin-triggered programmed cell death (PCD) by effector proteins. In this study, we constructed a PVX-*VmRnt2* vector and transformed it into *Agrobacterium tumefaciens* strain GV3101. Agroinfiltration of *N. benthamiana* leaves with *Agrobacterium* expressing VmRnt2 did not induce cell death **(**Fig. 2A**)**. Under the same conditions, cell death was elicited in leaves infiltrated with *Agrobacterium* expressing INF1. However, when VmRnt2 was co-expressed within INF1, it effectively suppressed the INF1-induced cell death **(**Fig. 2A**)**. Moreover, co-expression of the mutant VmRnt2^H80/H142A^ with INF1 abolished this suppression, thereby restoring the cell death phenotype **(**Fig. 2B**)**. Relative to the GFP control, leaves expressing VmRnt2 showed a reduced burst of reactive oxygen species (ROS), diminished ROS accumulation, and decreased callose deposition at 48 hpi (Fig. 2C–F). Additionally, detached leaves transiently overexpressing VmRnt2 developed larger lesions following inoculation with *Sclerotinia sclerotiorum* compared to those expressing GFP (Fig. 2G and H). In line with these findings, the expression of defense-related genes *NbWRKY7* and *NbPR1* was significantly downregulated in plants expressing VmRnt2 (Fig. 2I and J). Collectively, these results demonstrate that VmRnt2 suppresses plant immune responses and enhances susceptibility to *S. sclerotiorum* colonization in *N. benthamiana.*

**Figure 3:**
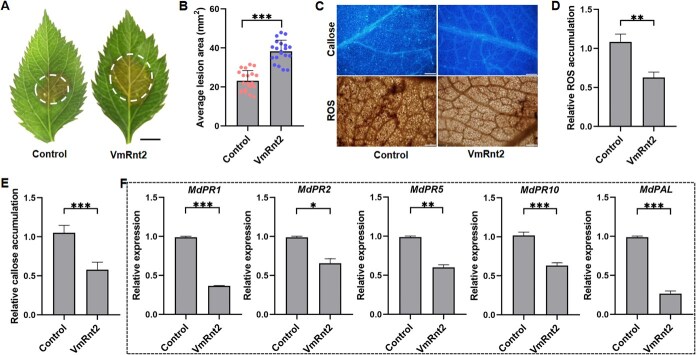
VmRnt2 enhances apple susceptibility to *V. mali.*  **(A and B)** Apple leaves lesion symptoms and lesion areas were assessed 72 h post-transient expression treatment with GFP (Control) and VmRnt2-GFP, and 36 hpi with *V. mali*. Scale bar represents 3 mm. **(C)** Deposition of callose and accumulation of ROS in apple leaves. Scale bar represents 100 μm. **(D, E)** The accumulation of ROS (D) and the deposition of callose (E) and in (C). **(F)** Relative expression levels of defense-associated genes *MdPR1*, *MdPR2*, *MdPR5*, *MdPR10*, and *MdPAL*. Leaves were sampled for RNA extraction at 3 d after Agro-infiltration of VmRnt2 and GFP. The internal reference gene MdActin was used for normalization. Relative expression of target genes was calculated relative to MdActin and calibrated to the buffer-treated control (assigned a value of 1). All data are expressed as mean ± standard deviation (*n* = 3). Statistical analyses were performed using Student’s *t*-test. Significant differences are indicated with asterisks (^*^P < 0.05, ^**^P < 0.01, ^***^P < 0.001).

To further explore the role of VmRnt2 in the defense mechanisms of apples, the construct pCAMBIA1302-*VmRnt2* was transiently overexpressed in apple cultivar Gala-3 seedlings using vacuum infiltration. Four days after infiltration, the leaves were inoculated with *V. mali*, and lesion areas were measured 24 hpi. The findings indicate that transient heterologous overexpression of *VmRnt2* diminishes the resistance of apples to *V. mali* compared to WT under standard growth conditions **(**Fig. 3A and B**)**. Additionally, this overexpression resulted in decreased ROS accumulation and callose deposition relative to the GFP control **(**Fig. 3C–E**)**. Concurrently, the expression levels of defense-related genes, including *MdPR1*, *MdPR2*, *MdPR5*, *MdPR10*, and *MdPAL*, were significantly reduced **(**Fig. 3F**)**. Taken together, these results suggest that heterologous expression of VmRnt2 impairs the immune responses of the plant, thereby increasing susceptibility to pathogens.

### VmRnt2 interacts with the apple TF MdMYB44

**Figure 4:**
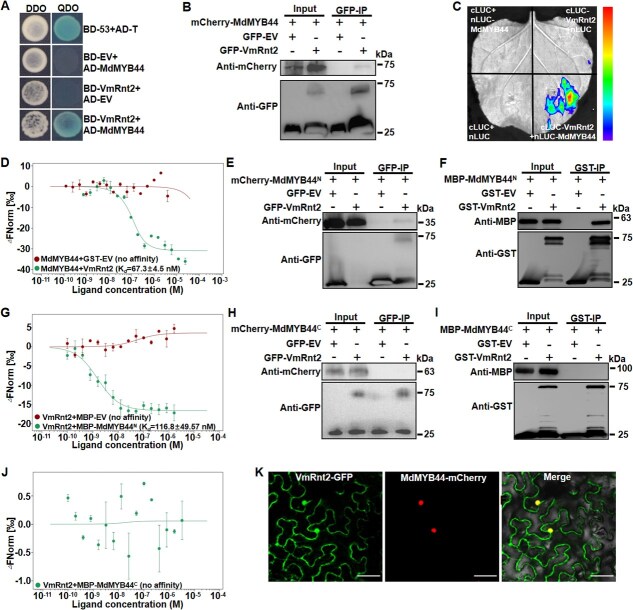
VmRnt2 interacts with the apple TF MdMYB44 in *vivo* and *vitro*. **(A)** Y2H assay showing the interaction between VmRnt2 and MdMYB44. BD-53 + AD-T served as positive control; BD + AD-MdMYB44 and BD-VmRnt2 + AD were used as negative control. DDO, SD/-Trp/-Leu; QDO, SD/-Trp/-Leu/-His/-Ade; BD, pGBKT7; and AD, pGADT7. **(B)**  *In vivo* Co-IP of VmRnt2 interacts with MdMYB44. Extracts from leaves co-expressing MdMYB44-mCherry and VmRnt2-GFP were immunoprecipitated with anti-GFP beads, and Western blotting were probed with anti-GFP or anti-mCherry antibodies. **(C)** Luciferase complementation imaging (LCI) demonstrating VmRnt2–MdMYB44 interaction. **(D)** Binding affinity between MdMYB44 and VmRnt2 were determined by microscale thermophoresis (MST) analysis. Labeled MdMYB44 was mixed with a gradient dilution of VmRnt2 recombinant protein. **(E)** Co-IP assays confirmed the interaction between MdMYB44^N^ and VmRnt2. **(F)** Pull-down assays confirmed the association between MdMYB44^N^ and VmRnt2. **(G)** Binding affinity between MdMYB44^N^ and VmRnt2 were determined by MST assays. **(H-J)** Co-IP, pull-down, and MST assays confirmed no detectable interaction between MdMYB44^C^ and VmRnt2. Data are expressed as mean ± standard deviation (*n* = 3). **(K)** Colocalization of VmRnt2-GFP and MdMYB44-mCherry in *N. benthamiana* leaves. Scale bar represents 40 μm.

Existing research has established that pathogen effectors undermine host immunity by targeting specific cellular components. In this context, we conducted a yeast two-hybrid (Y2H) screen using VmRnt2 as bait against a cDNA library from *V. mali*-infected apple, identifying 18 potential targets **(**Supplementary Table S2**)** that might mediate the functional role of VmRnt2 during infection. Our analysis confirmed that the apple TF MdMYB44 is a target of VmRnt2, as demonstrated by their interaction in a full-length Y2H assay **(**Fig. 4A**)**. Subsequent co-immunoprecipitation (Co-IP) assays in *N. benthamiana* leaves transiently co-expressing MdMYB44-mCherry and VmRnt2-GFP showed that MdMYB44-mCherry was detectable in the immunoprecipitate only when co-expressed with VmRnt2-GFP, and not in negative controls **(**Fig. 4B**)**. A split-luciferase complementation (Split-LUC) assay further confirmed strong interaction between VmRnt2 and MdMYB44 in *N. benthamiana*  **(**Fig. 4C**)***.* Additionally, microscale thermophoresis (MST) quantified the binding affinity, revealing a dissociation constant (Kd) of 67.3 ± 4.5 nM for MdMYB44-His/VmRnt2-GST interaction, while no binding was detected for the MdMYB44-His/GST control **(**Fig. 4D**)**.

To identify the specific interaction regions of VmRnt2 with MdMYB44, two recombinant constructs of MdMYB44 containing SANT domains N-terminus (1–111 aa) and C-terminus (112–319 aa) were constructed. Co-IP, pull-down, and MST assays showed VmRnt2 specifically interacted with the MdMYB44 N-terminus **(**Fig. 4E–G**)**, but not the C-terminus **(**Fig. 4H–J**)**, indicating the N-terminus SANT domain of MdMYB44 regions is essential for binding. The specific subcellular localization of VmRnt2–MdMYB44 interactions was further investigated through co-expression of fluorescently tagged proteins (VmRnt2-GFP and MdMYB44-mCherry) in *N. benthamiana*. Laser confocal microscopy revealed that VmRnt2 and MdMYB44 predominantly co-localized in the nuclei of *N. benthamiana* cells, demonstrating that their interaction occurs within the plant cell nucleus **(**Fig. 4K**)**. Collectively, these *in vivo* and *in vitro* results demonstrate a specific physical interaction between VmRnt2 and MdMYB44 that is mediated by the N-terminal region of MdMYB44.

### MdMYB44 positively modulates resistance to *V. mali*

To elucidate the role of MdMYB44 in the interactions between apple and *V. mali*, we first analyzed its expression during infection. qRT-PCR analysis revealed a significant induction of *MdMYB44* transcripts at 4 hpi in the incompatible interaction **(**Supplementary Fig. S6**)**. This early enhancement suggests that MdMYB44 plays a pivotal role in modulating the initial defense response of apple to *V. mali*, thereby contributing to resistance regulation.

To further assess the function of *MdMYB44* in disease resistance, we employed transient overexpression and silencing approaches. Ectopic expression significantly increased resistance in apple leaves to *V. mali* (Supplementary Fig. S7A and B), with overexpression confirmed via qRT-PCR (Supplementary Fig. S7C). Concurrently, the expression levels of defense-related genes, namely *MdPR1*, *MdPR2*, *MdPR10*, and *MdNPR1*, were significantly elevated in the *MdMYB44-*OE plants **(**Supplementary Fig. S7D**)**. Conversely, transient silencing of *MdMYB44* enhanced susceptibility to *V. mali*  **(**Supplementary Fig. S8A–C**)** and led to the downregulated of these defense genes **(**Supplementary Fig. S8D**)**. Subsequently, stable *MdMYB44-*overexpressing and -silencing apple calli were generated and verified through PCR **(**Supplementary Fig. S9A and B**)** and qRT-PCR (Supplementary Fig. S9C). Pathogen challenge assays indicated significantly increased resistance to *V. mali* in the overexpression lines compared to WT controls, whereas the silenced lines displayed heightened susceptibility **(**Supplementary Fig. S9D and E**)***.* These findings delineate MdMYB44 as a critical positive modulator of immunity in apple callus against *V. mali*.

**Figure 5:**
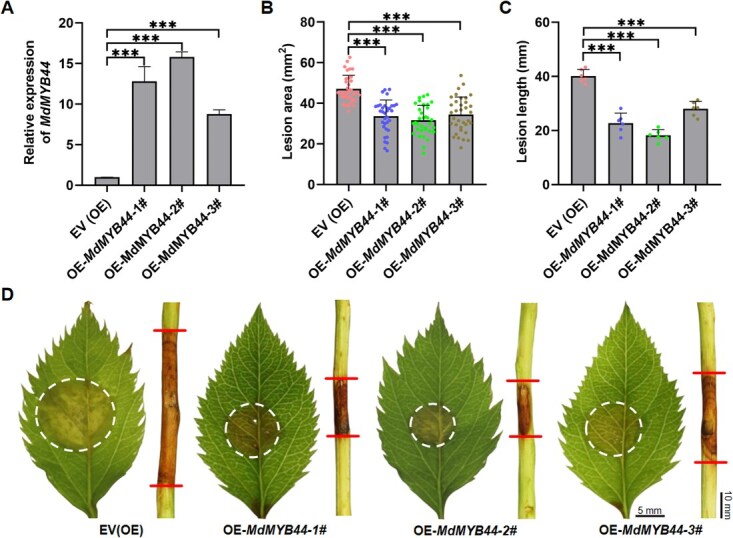
The stable overexpression *MdMYB44* (*MdMYB44*-OE) transgenic lines enhanced apple resistance to *V. mali*. **(A)** Relative expression levels of *MdMYB44* in *MdMYB44*-OE transgenic lines. **(B)** Lesion area of *MdMYB44*-OE transgenic lines apple leaves at 36 hpi. **(C)** Lesion length of *MdMYB44*-OE transgenic lines apple leaves at 72 hpi. Data are expressed as mean ± standard deviation (*n* = 6). **(D)** Lesion symptoms of the *MdMYB44*-OE transgenic lines after infection with *V. mali*. Scale bar represents 5 mm and 10 mm, respectively. Significant differences are indicated with asterisks (Student’s *t*-test, ^***^P < 0.001). All experiments were independently repeated three times with similar results.

To further explore the function of MdMYB44, we engineered stable overexpression lines in GL-3 seedlings. Three independent transgenic lines (OE-1, OE-2, OE-3) exhibited 12.5-, 15.0-, and 7.8-fold increases in *MdMYB44* transcripts, respectively (Fig. 5A). The successful establishment of these lines was further confirmed by PCR and protein abundance assays (Supplementary Fig. S10A and B). Disease evaluations demonstrated significantly smaller lesion areas on leaves of OEs (28.5%, 32.6%, and 26.8% reduction versus EV) and shorter lesion lengths on twigs (43.3%, 54.7%, and 30.1% reduction versus EV) (Fig. 5B–D). Collectively, these findings implicate MdMYB44 as a crucial positive regulator of the defense response in apple against *V. mali*.

### MdMYB44 transcriptionally activates *MdPR1A* expression through direct promoter binding, thereby enhancing the resistance of apple plants to *V. mali*

**Figure 6:**
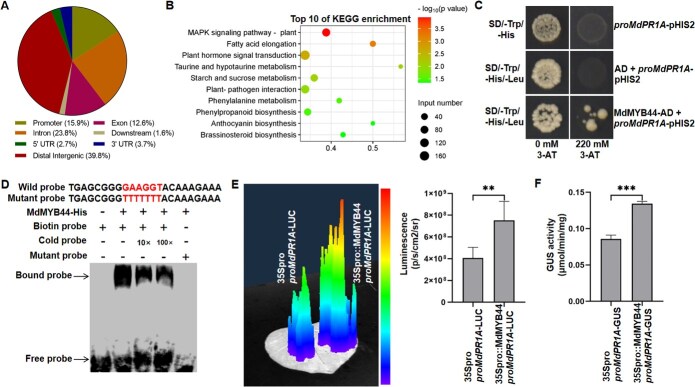
MdMYB44 binds to the promoter of *MdPR1A* to transcriptionally activate its expression. **(A)** Genome-wide distribution of MdMYB44 target genes identified by DAP-seq. **(B)** Top ten KEGG enrichment terms for genes bound by MdMYB44 in DAP-seq analysis. **(C)** Y1H assays demonstrating MdMYB44 binding to the *MdPR1A* promoter. **(D)** Electrophoretic mobility shift assay (EMSA) confirming the direct binding of MdMYB44 to the *MdPR1A* promoter. **(E)** Luciferase (LUC) reporter assays showing MdMYB44-dependent transcriptional activation of *MdPR1A*. **(F)** β-Glucuronidase (GUS) activity assays further verifying transcriptional upregulation of MdPR1A by MdMYB44. Data are expressed as mean ± standard deviation (*n* = 3). Significant differences are indicated with asterisks (Student’s *t*-test, ^**^P < 0.01, ^***^P < 0.001). All experiments were independently repeated three times with similar results.

As a TF, MdMYB44 is likely to modulate immune responses by regulating the expression of target genes. We utilized DNA affinity purification sequencing (DAP-seq) to identify genes regulated by MdMYB44. Analysis of binding peaks revealed that 15.9% were localized within the promoter regions **(**Fig. 6A**)**. Enrichment analysis using the Kyoto encyclopedia of genes and genomes (KEGG) indicated significant enrichment of target genes in pathways including the MAPK signaling pathway, fatty acid elongation, and plant hormone signal transduction, among others **(**Fig. 6B**)**. Given the role of MdMYB44 in conferring resistance to *V. mali* in apple plants, we specifically investigated genes in the MAPK signaling pathway that are regulated by MdMYB44. Several genes from the MAPK signaling pathway were selected for further analysis using yeast one-hybrid (Y1H) assays. The gene encoding the pathogenesis-related protein 1A (*MdPR1A*) in apple was identified as a potential target. In the Y1H assays, co-transformed plasmids containing MdMYB44-AD and pro*MdPR1A*-pHIS2 enabled yeast growth on SD/−Trp/−Leu/-His medium supplemented with 220 mM 3-AT, whereas controls containing only AD and pro*MdPR1A*-pHIS2 did not **(**Fig. 6C**)**, indicating that direct MdMYB44 directly binds to the *MdPR1A* promoter. To confirm this binding, we purified the MdMYB44 protein and conducted electrophoretic mobility shift assays (EMSA). The results showed specific binding of MdMYB44 to upstream regions of the *MdPR1A* promoter **(**Fig. 6D**)**. Further assessment of transcriptional regulation was performed using dual-luciferase assays in *N. benthamiana* leaves co-infiltrated with 35Spro::MdMYB44 and *proMdPR1A*-LUC constructs. The luminescence signal was significantly higher than that in the control (35Spro + *proMdPR1A*-LUC) **(**Fig. 6E**)**. Consistently, β-glucuronidase (GUS) activity and staining assays indicated stronger signals in leaves co-infiltrated with 35Spro::MdMYB44 and *proMdPR1A*-GUS compared to the control (35Spro + proMdPR1A-GUS) **(**Fig. 6F**;**  Supplementary Fig. S10C**)**, confirming that MdMYB44 functions as a transcriptional activator of *MdPR1A*.

Pathogenesis-related proteins (PRs) have been demonstrated to play critical roles in plant defense responses. Consequently, we hypothesized that MdMYB44 might confer resistance to *V. mali* in apple by modulating *MdPR1A* expression. To test this hypothesis, we quantitatively analyzed *MdPR1A* transcript levels in *MdMYB44*-OE and *MdMYB44*-RNAi apple calli. The results showed that a significant elevation of *MdPR1A* mRNA levels in *MdMYB44*-OE calli and marked suppression in *MdMYB44*-RNAi calli **(**Supplementary Fig. S10D**)**, demonstrating that MdMYB44 positively regulates *MdPR1A* expression.

**Figure 7:**
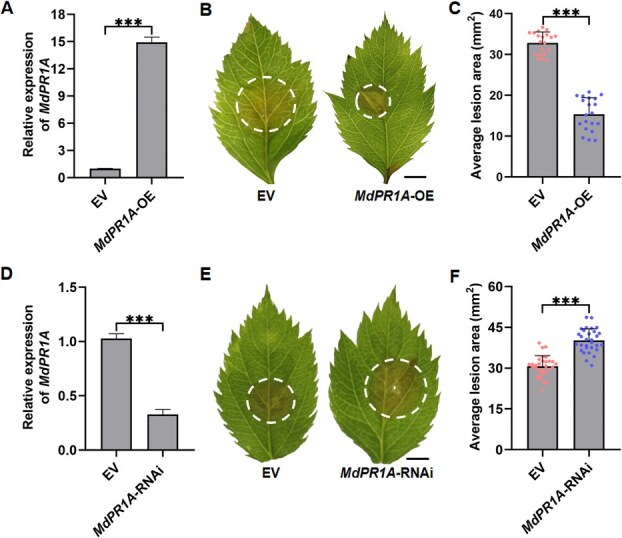
MdPR1A positively modulates resistance to *V. mali*. **(A)** Relative expression levels of *MdPR1A*. **(B)** Lesion symptoms on apple seedlings inoculated with *V. mali* following transient overexpression of *MdPR1A*. Scale bar represents 4 mm. **(C)** Lesion areas on apple leaves with transient *MdPR1A* overexpression at 36 hpi. **(D)** Relative expression levels of *MdPR1A*. **(E)** Lesion symptoms on apple seedlings inoculated with *V. mali* following transient silencing of *MdPR1A.* Scale bar represents 4 mm. **(F)** Lesion areas on apple leaves with transient *MdPR1A* silencing 36 hpi. The internal reference gene *MdActin* was used for normalization. Relative expression of target genes was calculated relative to *MdActin* and calibrated to the buffer-treated control (assigned a value of 1). Data are expressed as mean ± standard deviation (*n* = 3). Statistical analyses were performed using Student’s *t*-test. Significant differences are indicated with asterisks (^***^P < 0.001).

Subsequently, transient expression assays using *Agrobacterium*-mediated transformation were performed in apple seedlings to investigate the functional contribution of *MdPR1A* to resistance against *V. mali*. Disease evaluation revealed that *MdPR1A*-OE GL-3 leaves exhibited enhanced resistance to *V. mali* compared to WT **(**Fig. 7A–C**)**, while *MdPR1A*-RNAi significantly increased susceptibility **(**Fig. 7D–F**)**. Collectively, these results establish that *MdPR1A* positively regulates apple resistance to *V. mali*.

### VmRnt2 modulates MdMYB44 to prevent transcriptional activation of the defense-related gene *MdPR1A*

**Figure 8:**
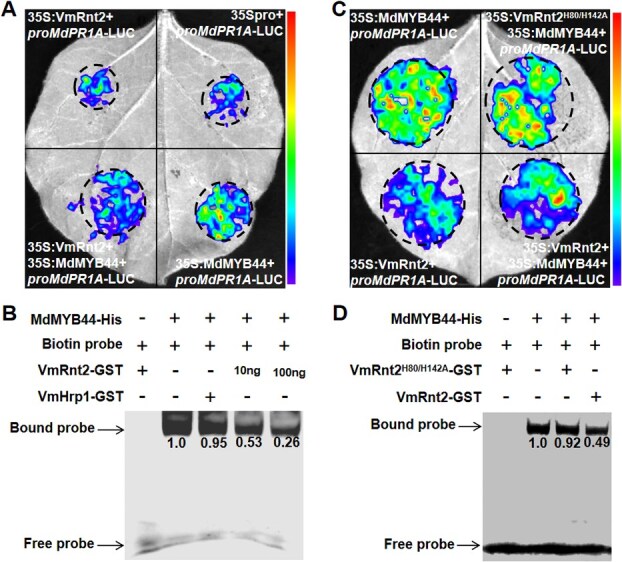
VmRnt2 interferes with the transcriptional activation activity of MdMYB44. **(A)** VmRnt2 expression inhibits the MdMYB44-activated *MdPR1A*. **(B)** EMSA assays confirmed that VmRnt2 inhibits the DNA-binding activity of MdMYB44. **(C)** VmRnt2^H80/H142A^ eliminated the inhibitory effect of VmRnt2 on the activation of *MdPR1A* by mdmyb44. **(D)** VmRnt2^H80/H142A^ restored the DNA binding activity of MdMYB44. MdMYB44-His was incubated with a biotin-labelled probe within the *MdPR1A* promoter and subjected to EMSA. VmRnt2-GST, VmRnt2^H80/H142A^-GST, and VmHrp1-GST (control) were, respectively, preincubated with MdMYB44-His for EMSA. All experiments were independently repeated three times with similar results.

Give that VmRnt2 inhibits immune responses in apple, we hypothesized that VmRnt2 might interfere with MdMYB44 binding to its downstream target genes the promoters. To test this hypothesis, we first evaluated the impact of VmRnt2 on MdMYB44 transcriptional activity using luciferase (LUC) reporter assays in *N. benthamiana*. The results demonstrated that the co-expression of 35Spro and *proMdPR1A*-LUC yielded luminescent signals comparable to those from the co-expression of 35Spro::VmRnt2 and *proMdPR1A*-LUC **(**Fig. 8A**)**, indicating that VmRnt2 alone does not affect *MdPR1A* transcription. However, luminescence significantly increased when 35Spro::MdMYB44 and pro*MdPR1A*-LUC were co-expressed; this activation was substantially reduced by the addition of 35Spro::VmRnt2 **(**Fig. 8A**)**. These findings suggest that VmRnt2 specifically inhibits the MdMYB44-mediated transcriptional activation of *MdPR1A*. In an EMSA, MdMYB44-His specifically bound to the promoter of *MdPR1A* (Fig. 8B). Notably, competitive EMSA revealed that preincubation with VmRnt2 substantially diminished the DNA-binding activity of MdMYB44 **(**Fig. 8B**)**, indicating a direct antagonistic mechanism by which VmRnt2 disrupts MdMYB44 transcriptional activity. Moreover, mutation of the catalytic site in VmRnt2 abolished its inhibition of MdMYB44-mediated transcriptional activation and concomitantly restored the DNA-binding ability of MdMYB44 **(**Fig. 8C and D**)**. This competitive inhibition suggests that VmRnt2 acts as a virulence effector to subvert host defense signaling by targeting transcription TF–DNA interactions essential for *MdPR1A*-mediated immunity.

## Discussion

Several studies have elucidated the role of fungal RNase T2 proteins as virulence effectors that undermine host defenses [31, 32]. For instance, *Ustilago maydis* secretes enzymes Nuc1 and Nuc2, both members of the RNase T2 family, to both facilitate pathogenicity and utilize extracellular RNA as a nutrient source [33]. Similarly, *F. oxysporum* strains FoRnt2 and FocRnt2 possess RNase activity that degrades host RNA *in vitro*, thereby augmenting fungal virulence [31, 34]. In this study, we cloned and characterized a secreted RNase protein, VmRnt2, which demonstrated high expression levels during the infection process of *V. mali*  **(**Fig. 1A**)**. Notably, pathogenicity toward apple hosts was significantly reduced following gene knockout, underscoring the pivotal role of VmRnt2 in the pathogenicity of *V. mali*  **(**Fig. 1D-F**)**.

Cell death serves as a crucial defense mechanism within plant immunity against pathogenic attacks [35]. For example, the protein VmHrp1, an elicitor from *V. mali*, robustly induces cell death and activates plant immune responses [36]. In a parallel vein, RNase Fg12 elicits cell death in *N. benthamiana* and bolsters resistance against various hemibiotrophic pathogens [37]. In contrast, transient expression of VmRnt2 in *N. benthamiana* did not induce detectable cell death **(**Fig. 2A**)**, aligning with observations that FoRnt2 from *F. oxysporum* f. sp. *lycopersici* similarly does not trigger cell death [31]. Additionally, we discovered that VmRnt2 inhibited INF1-induced cell death **(**Fig. 2A**)** and significantly heightened the susceptibility of *N. benthamiana* to *S. sclerotiorum* infection **(**Fig. 2G and H**)**. Remarkably, transient overexpression of VmRnt2 in apple substantially increased *V. mali* virulence **(**Fig. 3A and B**)**. The critical roles of effectors from phytopathogenic fungi in suppressing plant defenses and facilitating fungal infection are well documented. For example, the effector VmSpm1 suppresses BAX-induced cell death in *N. benthamiana* and is essential for achieving full virulence in *V. mali* [27]*.* Additionally, VmRnt2 significantly downregulated defense-related genes in both *N. benthamiana* (*NbWRKY7* and *NbPR1*) **(**Fig. 2I and J**)** and apple (*MdPR1*, *MdPR2*, *MdPR5*, *MdPR10*, and *MdPAL*) **(**Fig. 3F**)**. These findings collectively identify VmRnt2 as a critical virulence factor that subverts plant immunity across both nonhost and host–pathogen interactions, thereby facilitating successful infection by suppressing PCD and basal defense responses.

TFs, particularly those belonging to the MYB family, play a pivotal role in regulating gene expression related to plant defense mechanisms, making them frequent targets of pathogen effectors. These effectors adopt a variety of strategies to inhibit TF-mediated resistance in the host [10]. Our research has shown that the *V. mali* effector, VmRnt2, physically interacts with the MYB TF MdMYB44 **(**Fig. 3**)**. Functional assessments indicated that overexpression of MdMYB44 enhances the resistance of apple trees to *V. mali*  **(**Fig. 5; Supplementary Fig. S7**)***.* In contrast, silencing MdMYB44 not only increases susceptibility to *V. mali* but also significantly reduces the expression of defense-related genes **(**Supplementary Fig. S8**)**. Utilizing DAP-seq, we identified numerous potential target genes under the regulation of MdMYB44. Experimental validations further confirmed that MdMYB44 binds to the promoter of the pathogenesis-related gene *MdPR1A*, thereby activating its expression **(**Fig. 6C-F**)**. Collectively, these findings confirm the role of MdMYB44 as a crucial positive regulator of resistance in apples to AVC, primarily through the transcriptional activation of defense-related genes.

**Figure 9:**
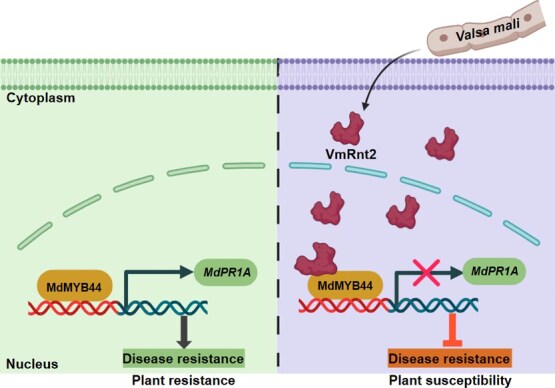
Proposed model demonstrating the role of VmRnt2 in regulating plant immunity. During infection by *V. mali* in apple, the effector protein VmRnt2 is secreted and translocated into the host cell nucleus, where it interacts with the apple TF MdMYB44. This interaction inhibits the transcriptional activation of the defense-related gene *MdPR1A*, thereby enhancing apple susceptibility to *V. mali.*

Pathogenesis-related proteins constitute a diverse group of proteins that are synthesized in response to pathogen recognition or defense signaling molecules [38, 39]. These proteins generally operate downstream in defense cascades, with functions that include degrading pathogen cell walls, inhibiting proteases, and producing defensins [40]. For example, OsWRKY114 in rice significantly increases resistance to *Xanthomonas oryzae* pv. *oryzae* (*Xoo*) by transcriptionally activating *OsPR1a* and modulating other defense-related genes [41]. Our findings reveal that overexpression of *MdMYB44* substantively elevates *MdPR1A* transcript levels, while its silencing diminishes these levels in apple leaves **(**Supplementary Fig. S10D**)**, thereby directly regulating *MdPR1A* transcription. Prior research has recognized PR1 transcript accumulation as a reliable molecular marker for the activation of salicylic acid (SA)-mediated defense signaling [42]. In this study, we demonstrated that MdMYB44 functions as a transcriptional activator of *MdPR1A*, implicating MdMYB44 in regulating apple resistance to *V. mali* via SA-dependent pathways. These results outline a transcriptional network mediated by the MYB family that confers pathogen resistance by coordinating SA-dependent immunity. This network effectively links transcriptional regulation with defense responses. In addition, pull-down and MST assays confirmed that VmRnt2 specifically interacts with the N-terminal SANT domain of MdMYB44 **(**Fig. 4F and G**)**. This competitive binding is likely to occlude the DNA-binding interface of MdMYB44 or alter the spatial configuration necessary for its association with DNA, thereby suppressing the activation of *MdPR1A* and downstream immune responses (Fig. 9).

In plants, MYB44 TF function as central integrators of defense signaling, coordinating responses to both biotic and abiotic stresses [43]. For example, AtMYB44 promotes PAMP-triggered immunity by upregulating EIN2 and MPK3/6, modulates insect resistance via glucosinolate pathways, and enhances resistance to bacterial wilt by activating spermidine synthase [44, 45]. In pear, a PcMYB44-mediated module confers resistance to ring rot disease through promoting lignification and activating the expression of disease-resistance genes involved in JA/SA/ET defense pathways [46]. In the present study, VmRnt2 suppresses MdMYB44 may impairing multiple downstream defense layers-including ethylene signaling, PTI response, and the biosynthesis of key antimicrobial compounds. This strategy effectively disrupts the host’s immune network and promotes the infection of pathogens. These findings establish VmRnt2 as a master effector molecule deployed by *V. mali* to systemically compromise host immunity. In summary, this study not only enhances our understanding of the mechanisms underlying plant–pathogen interactions but also provides essential insights into resistance-associated gene networks and transcriptional regulatory mechanisms. Our findings lay the groundwork for the development of disease-resistant crop varieties through the strategic manipulation of SA signaling pathways.

## Materials and methods

### Microbial strains and plant growth conditions

Strains of *V. mali* (WT, *ΔVmRnt2* mutant, and complemented strain *ΔVmRnt2-C*) were cultured on PDA at 25°C in complete darkness. Tissue-cultured seedlings of GL-3 apple (*Malus domestica*) were grown on Murashige and Skoog (MS) basal medium supplemented with 0.2 mg/L 6-benzylaminopurine (6-BA) and 0.2 mg/L indole-3-acetic acid (IAA). ‘Orin’ apple calli were cultivated under dark conditions at 25°C on MS medium, which was enriched with 0.4 mg/L 6-BA and 1.5 mg/L 2,4-dichlorophenoxyacetic acid (2,4-D), adhering to the protocols described by Han et al. [47]. *N. benthamiana* plants were cultivated in a greenhouse, maintaining a 16-h light/8-h dark photoperiod at 25°C.

### RNA extraction and qRT-PCR assays

Total RNA was isolated using an RNA Isolation Kit (Sangon Biotechnology, Shanghai, China) as per the manufacturer’s guidelines. First-strand cDNA synthesis was performed from 2 μg total RNA utilizing the RevertAid First Strand cDNA Synthesis Kit (Thermo Scientific, USA). qRT-PCR was executed on a LightCycler 96 System (Roche, Basel, Switzerland) employing 2 × RealStar Fast SYBR qPCR Mix (GenStar, Beijing, China). The genes *NbActin* and *MdActin* served as internal controls for normalizing gene expression in *N. benthamiana* and *M. domestica*, respectively. Expression levels were quantified via the 2^−*ΔΔ*Ct^ method. Each experimental setup included three biological replicates. Primer sequences are available in Supplementary Table S1.

### Transformant generation and pathogen inoculation

The *VmRnt2* gene knockout mutant was generated employing a double-joint PCR method. Primers detailed in Supplementary Table S1 were used to amplify sequences flanking the target region for constructing the deletion cassette. The resulting constructs were purified and introduced into *V. mali* strain 03–8 protoplasts using PEG-mediated transformation [48]. A schematic of the gene deletion strategy and verification of transformants is depicted in Supplementary Fig. S1**.** Genomic PCR, utilizing primers from Supplementary Table S1, screened for positive transformants. For virulence assays, detached apple twigs were inoculated with the WT *V. mali*, the *ΔVmRnt2* mutant, or the complemented strain*ΔVmRnt2-C*. In *N. benthamiana*, 48 h after infiltration with *A. tumefaciens* cells harboring the full-length VmRnt2 (with SP), fresh mycelial plugs (5 mm in diameter) were placed on the center of the leaves. Plants were then maintained in high-humidity chambers, and lesion areas caused by *S. sclerotiorum* were measured at 36 hpi.

### Yeast signal sequence trap

The predicted SP coding sequence of VmRnt2 was inserted into pSUC2 vector, resulting in the construct pSUC2-SP^VmRnt2^. This construct, along with a positive control (pSUC2-Avr1b) and a negative control (pSUC2-Mg87), was introduced into the invertase-deficient yeast strain YTK12. Transformants were selected on CMD-W medium. Positive colonies were subsequently cultured on YPRAA medium, to promote the secretion of invertase. Following the protocol established by Nie et al. [48], SP^VmRnt2^ was fused to the N-terminus of the INF1 elicitin, which lacks its native SP (SP^VmRnt2^-INF1^*Δ*SP^). This fusion protein was transiently expressed in *N. benthamiana* to evaluate the SP by monitoring the cell death response.

### Agroinfiltration assays

*A. tumefaciens* strains harboring either the PVX-VmRnt2 or the PVX-VmRnt2^*Δ*SP^ constructs were resuspended in an infiltration buffer containing 10 mM magnesium chloride, 0.5 mM MES, and 0.2 mM acetylacetone, adjusted to a final OD600 of 0.6, and infiltrated into the leaves of 4-week-old *N. benthamiana* plants. PCD was evaluated at 5 days post-infiltration (dpi). For co-localization analysis, coding sequences for VmRnt2-GFP and MdMYB44-mCherry were individually cloned into the vectors pCAMBIA1302-GFP and pICH86988-mCherry, respectively. The resulting constructs, pCAMBIA1302-VmRnt2-GFP and pICH86988-MdMYB44-mCherry, were transformed into *Agrobacterium* GV3101. Bacterial suspensions, adjusted to an OD_600_ of 0.6, were co-infiltrated into *N. benthamiana* leaves. Fluorescence was visualized at 2 dpi using an Olympus FV3000 laser scanning confocal microscope (Olympus Corp., Tokyo, Japan).

### Trypan blue staining

PCD in *N. benthamiana* leaves was examined using trypan blue staining. Leaf sections collected for PCD verification were boiled and stained in a 1 g/L trypan blue solution (glycerin: lactic acid: phenol: ethanol = 1:1:1:7) for 5 min, followed by incubation in darkness for 12 h. The samples were then decolorized in a 1.25 g/L chloral hydrate solution for 48 h to remove background staining. The decolorized samples were subsequently photographed to document the results.

### ROS burst measurement, ROS detection, and callose staining

Leaf discs were excised from *N. benthamiana* leaves expressing VmRnt2. These discs were placed in a 96-well plate and incubated overnight in 100 μL of distilled water. Subsequently, the water was replaced with a reaction solution comprising 100 μM luminol (Solarbio, Beijing, China), 20 μg/ml horseradish peroxidase, and an elicitor (either 8 μM hexaacetylchitohexaose or water as a control). Luminescence was quantified using a Varioskan LUX multimode microplate reader (Thermo Scientific). For ROS staining, tobacco and apple leaves infiltrated with *A. tumefaciens* harboring VmRnt2 were harvested. These were then immersed in a 1 mg/ml 3,3′-diaminobenzidine (DAB) solution (pH 3.8) and incubated overnight under light conditions. Following this, leaves were decolorized in 95% ethanol at 56°C for 3 h to ensure complete chlorophyll removal. For callose staining, leaves (either following the DAB staining protocol or similarly processed) were stained for callose deposition. Samples were incubated in a toluidine blue staining solution (0.01% aniline blue in 150 mM K_2_HPO_4_ buffer) in the dark for 12 h. Callose deposits were visualized under UV epifluorescence illumination using an Olympus microscope. Photographs were taken for analysis. Quantification of ROS accumulation (based on DAB staining intensity) and callose deposition was performed using ImageJ software according to the following procedure. The images were first decomposed into grayscale and the objects to be tested were adjusted to red. The fluorescence intensity of the red areas was measured [49].

### Ribonuclease activity assays

*In vitro* RNase assays were conducted to evaluate the enzymatic function of VmRnt2 against total RNA extracted from apples [31]. Total apple RNA (5 μg) was incubated for 30 min at 25°C with either purified GST (as a tag control), VmRnt2 protein, or the catalytic-site mutant VmRnt2^H80/H142A^. Following treatment, samples were mixed with 1 × loading buffer and subjected to electrophoresis on a 1% agarose gel.

### Yeast two-hybrid assays

To screen *V. mali*-apple interact library, a bait construct was engineered by cloning the coding region of VmRnt2 (lacking its SP) in-frame into pGBKT7. Yeast transformants were initially selected on DDO medium (SD/-Leu/-Trp). Candidate positive transformants were subsequently transferred to QDO medium (SD/-Leu/-Trp/-His/-Ade) and incubated at 28°C for three days to confirm protein–protein interactions.

### Split-LUC assay

Split-LUC assays were conducted in accordance with the methods described by Dong *et al*. [50]. In brief, the coding sequences of *MdMYB44* and *VmRnt2* were cloned into the pCAMBIA1300-nLuc and pCAMBIA1300-cLuc vectors, respectively, to generate the fusion constructs pCAMBIA1300-MdMYB44-nLuc and pCAMBIA1300-VmRnt2-cLuc. These recombinant vectors were then transformed into the *A. tumefaciens* strain EHA105 and co-infiltrated into *N. benthamiana* leaves in various combinations. Between 48 and 72 hpi, 1 mM D-luciferin was administered to the infiltrated areas. Following a 5-min period of dark adaptation and luciferase complementation signals were detected.

### Co-IP assays

The relevant constructs were co-transfected into *N. benthamiana* via *A. tumefaciens*-mediated transient expression. Agroinfiltrated leaf tissues were collected at 48 hpi. Total protein was extracted from 500 mg of powdered tissue using 1 ml of native lysis buffer, which was supplemented with 1 mM phenylmethylsulfonyl fluoride (PMSF) and 1% proteinase inhibitor cocktail (Solarbio, Beijing, China). Following centrifugation at 15 000 × *g* for 20 min at 4°C, the supernatant was collected and incubated with 20 μl of GFP-Trap® Magarose beads (Smart-Lifesciences, Cat. No. SM038001, Changzhou, China) at 4°C for 3 h with constant rotation. The beads were then washed three times with the lysis buffer. Proteins bound to the beads were eluted and separated by 10% SDS-PAGE, followed by immunoblot analysis using anti-GFP (1:5000, Abmart, M20004, Shanghai, China) or anti-mCherry (1:2000, Proteintech, 26 765-1-AP, Chicago, IL, USA) antibodies.

### MST assays

Recombinant MdMYB44-His protein was fluorescently labeled using the Monolith Protein Labeling Kit (NanoTemper Technologies, Germany) following the manufacturer’s instructions. The labeled proteins were then incubated with serial dilutions of recombinant VmRnt2 protein for 30 min at room temperature. The binding reactions were subsequently transferred to premium capillaries (NanoTemper) and analyzed on a Monolith NT.115 instrument via MST. Binding affinity was calculated from three independent replicates using the MO.Affinity Analysis software v2.3.0.

### Y1H assays

The coding sequence of MdMYB44 and the promoter sequence of *MdPR1A* were respectively cloned into the pGADT7 vector and the *proMdPR1A*-pHIS2 vector. To mitigate self-activation of the reporter system, a concentration of 160 mM 3-amino-1, 2, 4-triazole (3-AT) (Coolaber, Beijing, China) was incorporated into the selection medium. The yeast strain Y187 was utilized for the Y1H assays.

### Dual-luciferase transient expression assays

Dual-luciferase assays were performed in accordance with the methodology previously described by Dong *et al*. [50]. In brief, the coding sequences of MdMYB44 were inserted into the pGreenII 62-SK vector to create the construct 35Spro::MdMYB44. The promoter sequences of *MdPR1A* were amplified and cloned into the pGreenII 0800-LUC vector, resulting in the reporter construct *proMdPR1A*-LUC. The empty pGreenII 62-SK vector (35Spro) served as a negative control. All constructs were introduced into *Agrobacterium* GV3101. Bacterial suspensions containing the constructs were mixed in the appropriate ratios and co-infiltrated into *N. benthamiana* leaves. For LUC imaging, infiltrated leaf areas were sprayed with 1 mM D-luciferin potassium salt, and luminescence signals were acquired using a PlantView100 fluorescence imaging system. Each experimental condition was independently replicated at least three times.

### GUS activity assay

For the GUS activity analysis, the constructs 35Spro::MdMYB44 (as generated above) and *proMdPR1A-GUS* (created by cloning the *MdPR1A* promoter into pCB308) were used. These constructs were transformed into *A. tumefaciens* strain GV3101. Bacterial suspensions containing the constructs were co-infiltrated into *N. benthamiana* leaves. Agroinfiltrated leaves were harvested at 48 hpi. GUS activity was quantified using a commercial GUS detection kit (Coolaber) following the manufacturer’s protocol.

### DAP-seq and EMSA assays

DAP-seq assays were conducted by Bluescape Hebei Biotechnology Co., Ltd. (Hebei, China). We identified putative target genes through a comparative analysis of high-throughput sequencing datasets against genome annotation databases.

EMSA were performed using a chemiluminescence EMSA kit (Beyotime Biotechnology, China), following the methodology outlined by Han *et al*. [25]. The MdMYB44-His fusion protein was expressed in *E. coli* and subsequently purified. Biotin-labeled DNA probes, synthesized by Sangon Biotechnology Co., Ltd. (Shanghai, China), were incubated with the purified protein. The resulting protein–DNA complexes were separated on 6% nondenaturing polyacrylamide gels, transferred to nylon membranes, and detected through chemiluminescence. To confirm the specificity of binding, competition assays were conducted using unlabeled probes (cold competitors). The primers used for the probe synthesis are detailed in Supplementary Table S1. All experiments were replicated independently three times.

### Genetic transformation assays

For the genetic transformation assays, three different vector systems pK2GW7-HA, pK7GWIWG2D (II)-GFP, and pCAMBIA1302-GFP were utilized [47, 50]. *Agrobacterium*-mediated transformation was employed to generate stable transgenic apple plants and calli. Transient expression experiments were conducted in 4-week-old GL-3 seedlings using the GV3101 (pSoup-P19) strain, while stable transformation was performed on GL-3 seedlings and 15-day-old calli using the EHA105 strain, as described by Han *et al*. [47].

### Accession numbers

The corresponding DNA and protein sequences from this study can be accessed via GenBank (https://www.ncbi.nlm.nih.gov/) using the provided accession numbers: VmRnt2 (KUI72745.1), MdMYB44 (NP_001287808.1), and MdPR1A (XP_008382391.1).

## Supplementary Material

Web_Material_uhag054

## Data Availability

All relevant data can be found within the published article and its supporting material.
