# Supplementary material for: The fungi effector VmRnt2 from Valsa mali modulates host transcription factor to suppress immunity in apple
Source: Hortic Res. 2026 Feb 26;13(6):uhag054. doi: 10.1093/hr/uhag054 (PMC13242955; doi:10.1093/hr/uhag054)

Supplemental Fig. S1

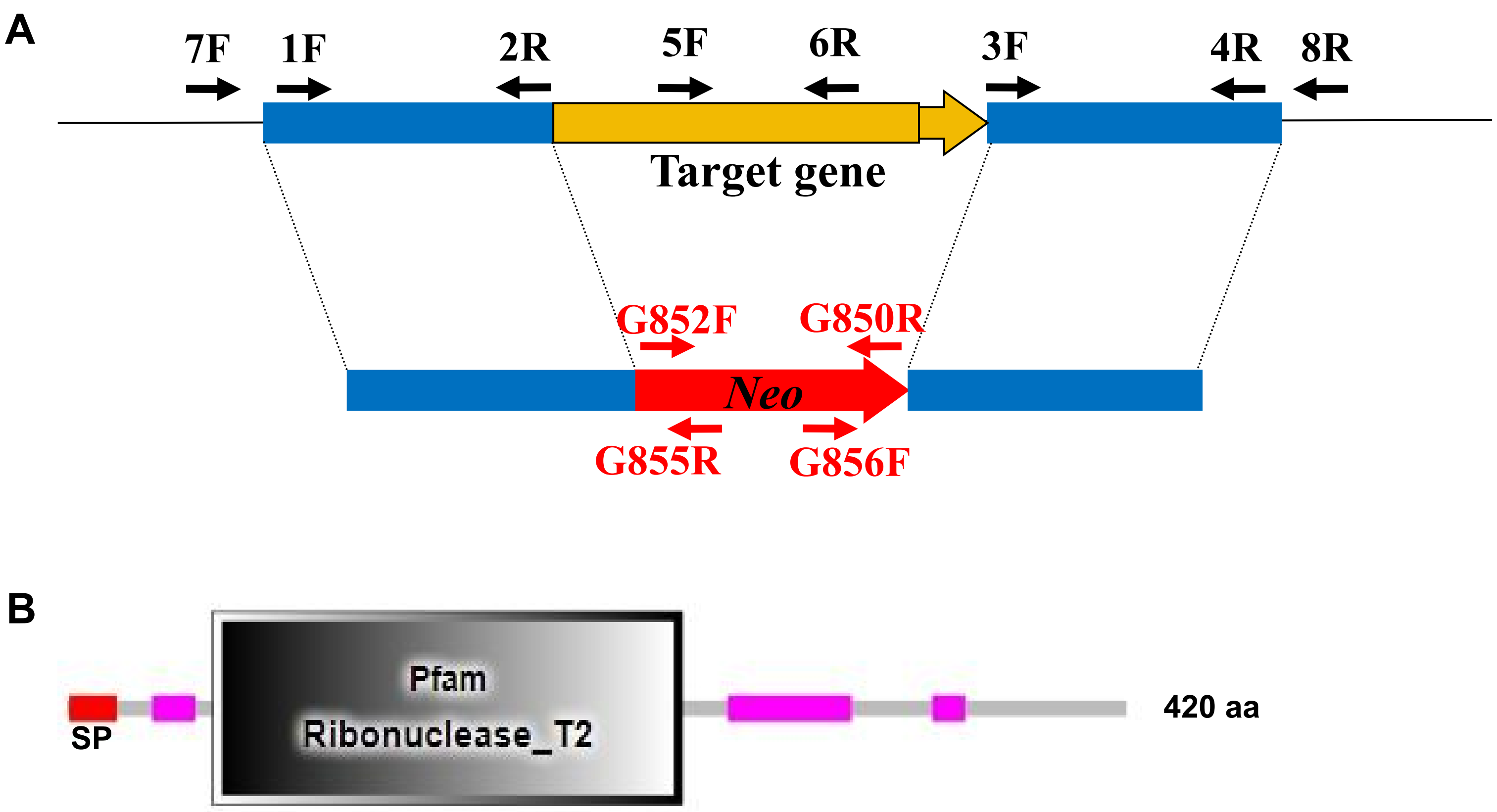

Supplemental Fig. S2

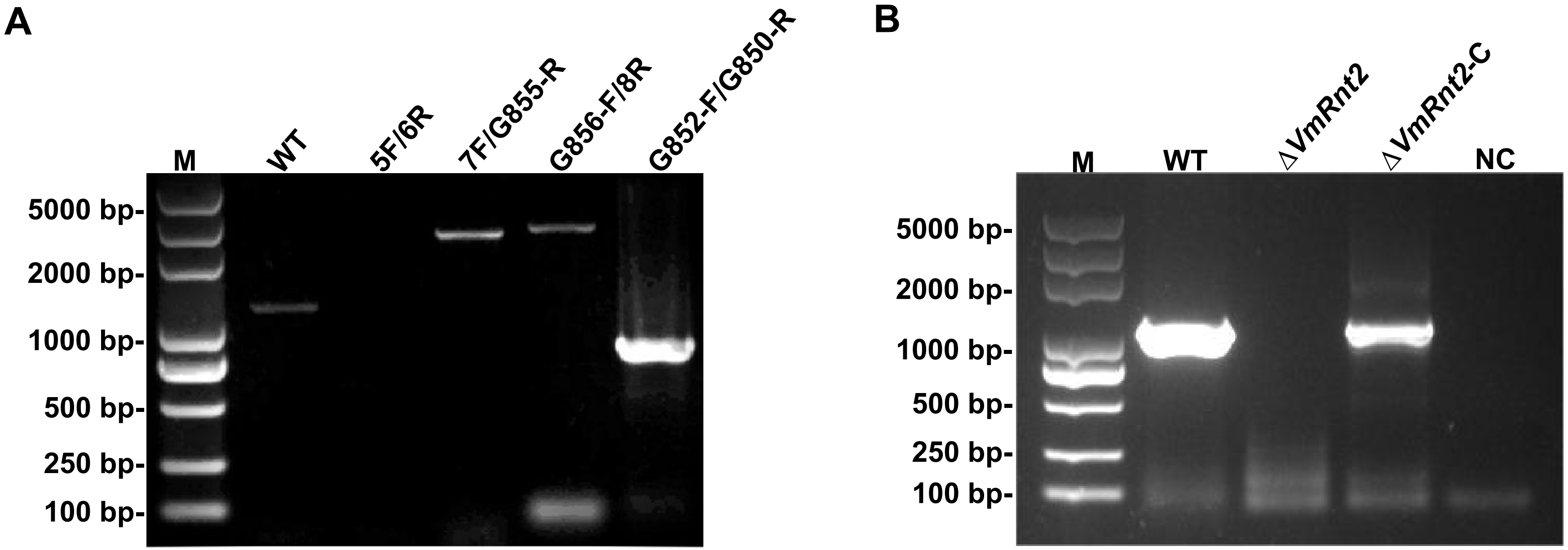

Supplemental Fig. S3

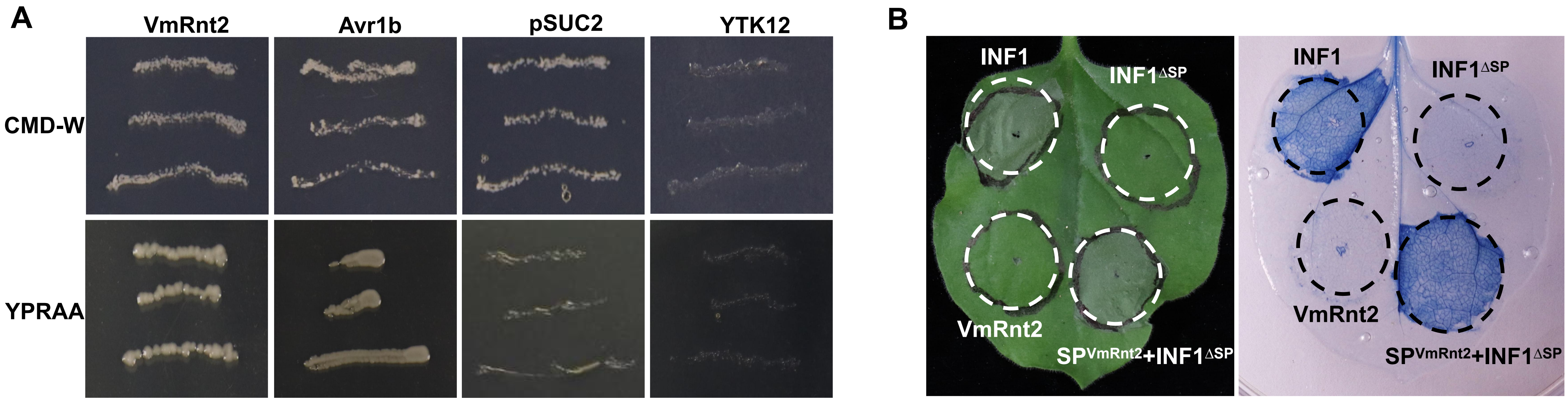

Supplemental Fig. S4

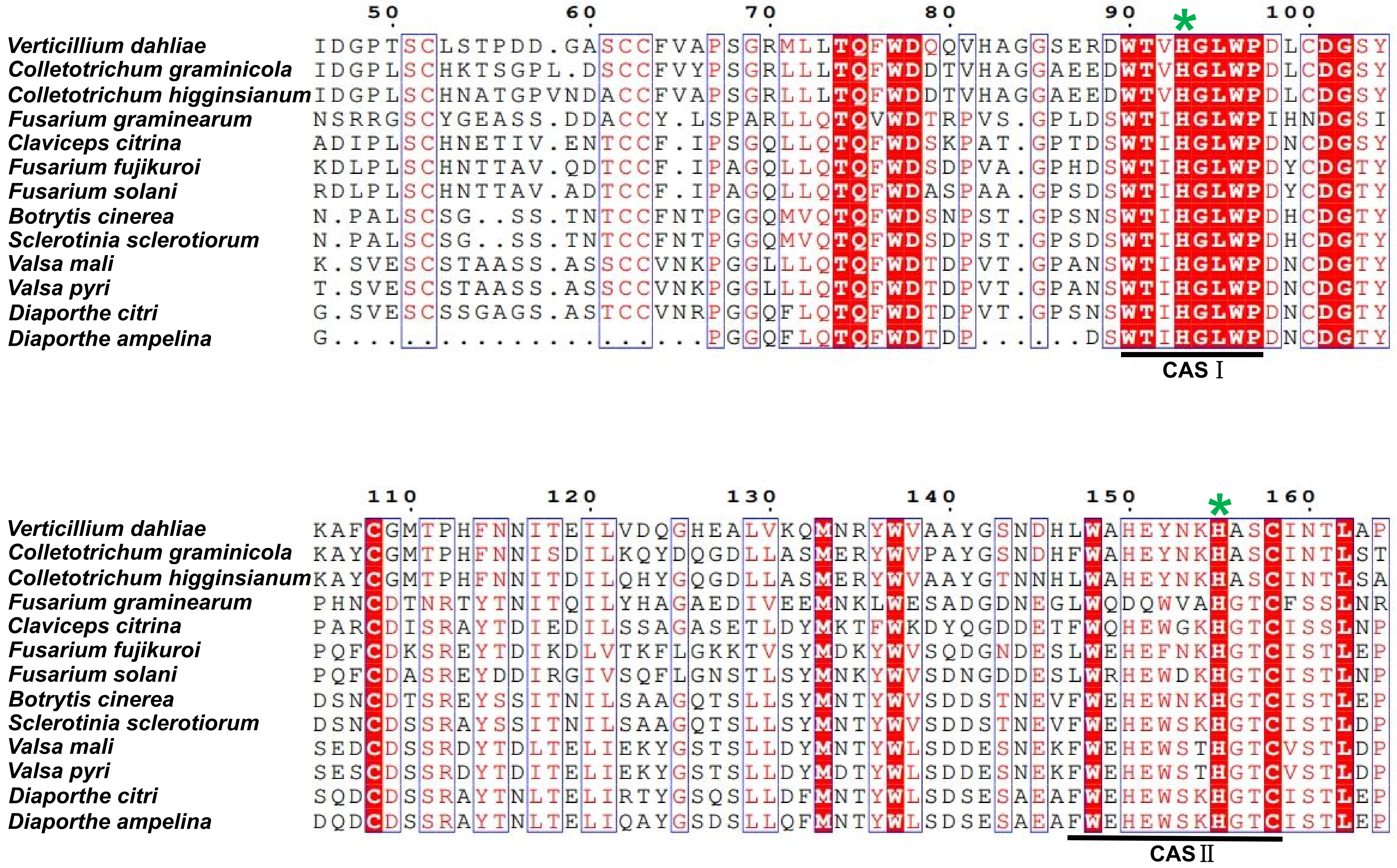

Supplemental Fig. S5

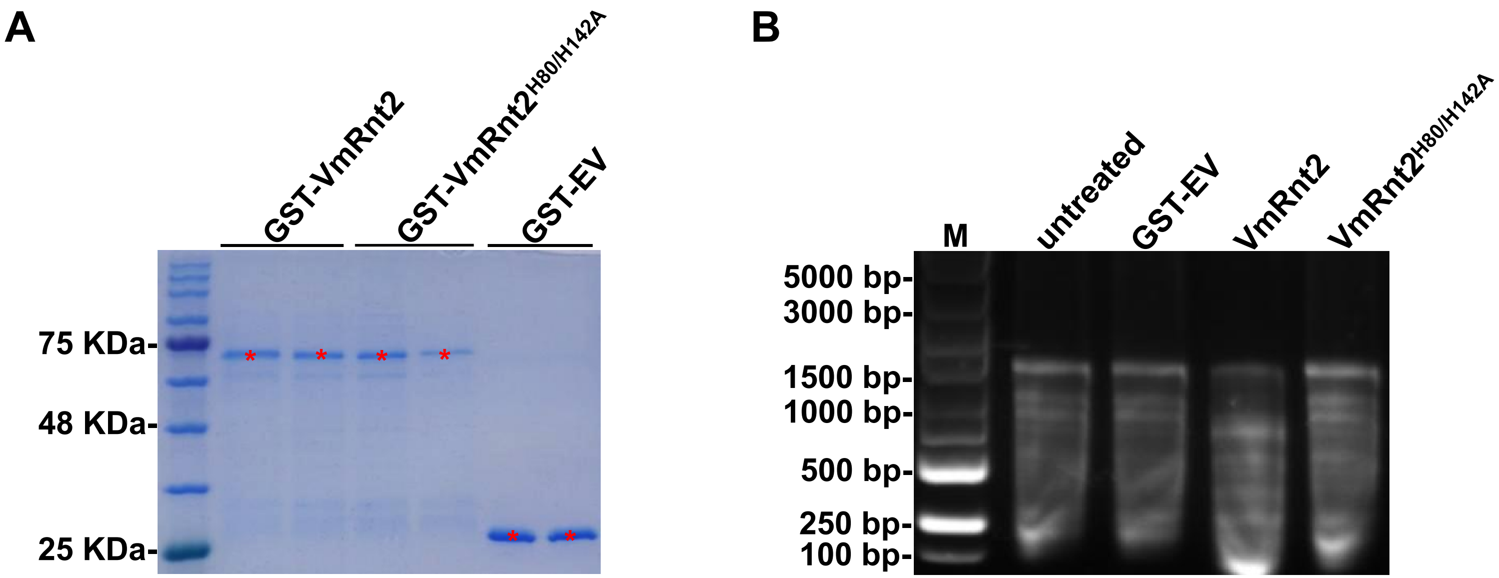

Supplemental Fig. S6

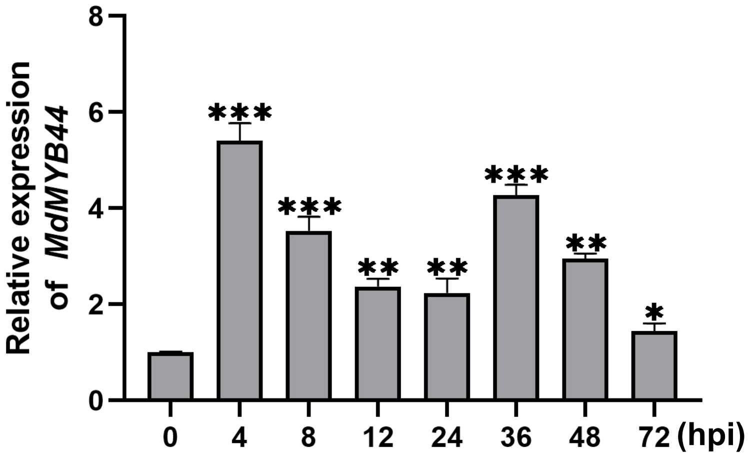

Supplemental Fig. S7

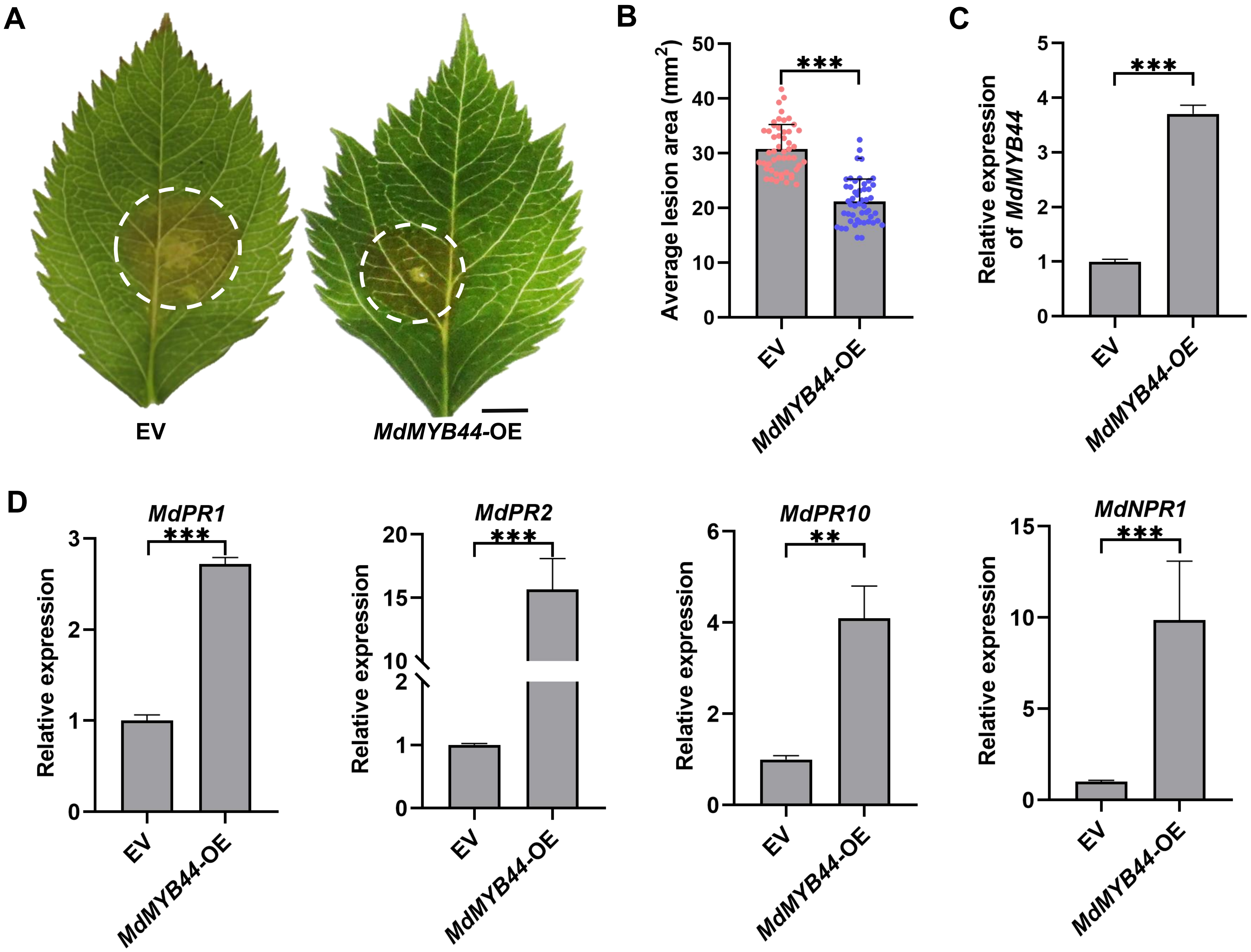

Supplemental Fig. S8

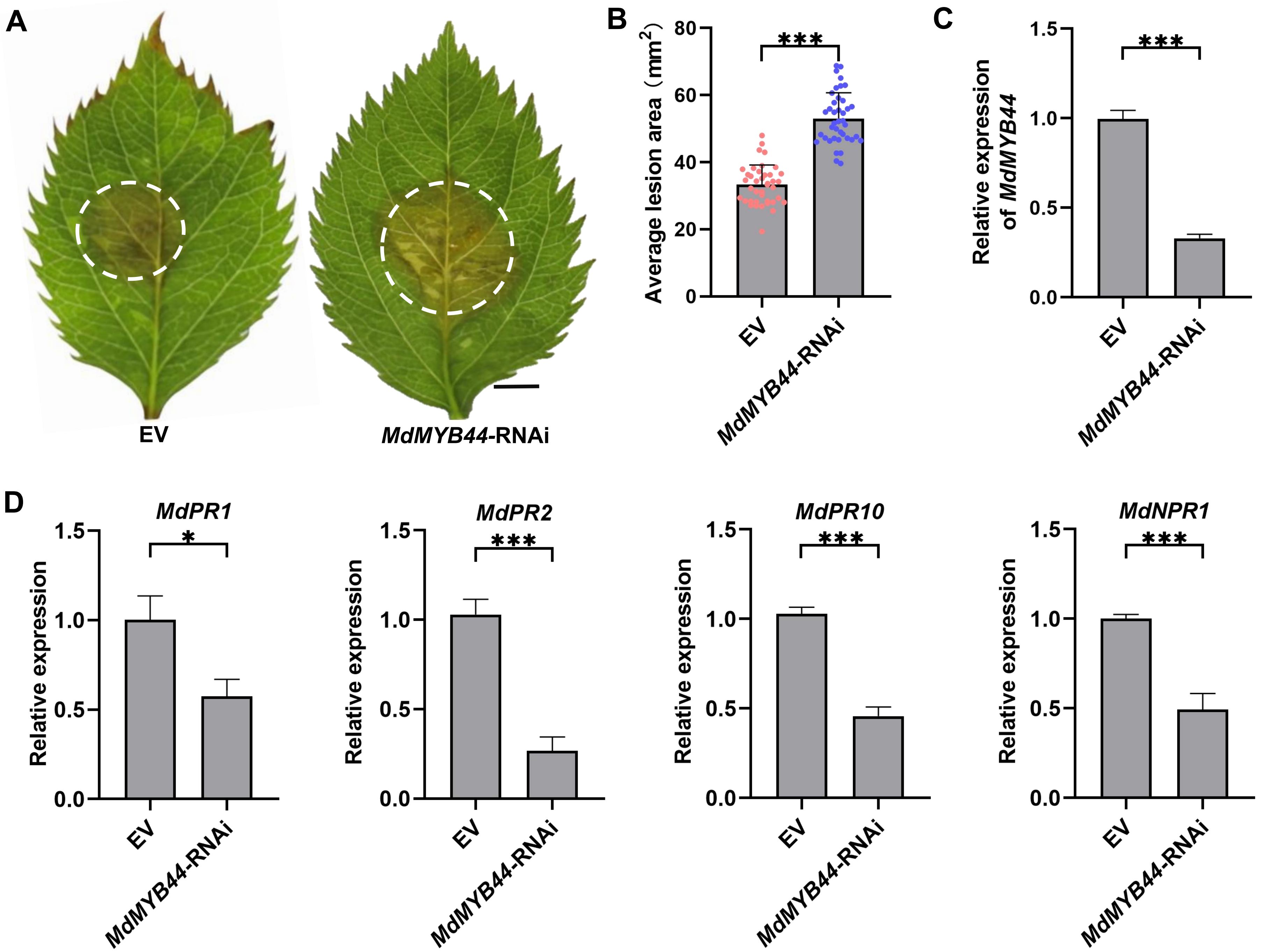

Supplemental Fig. S9

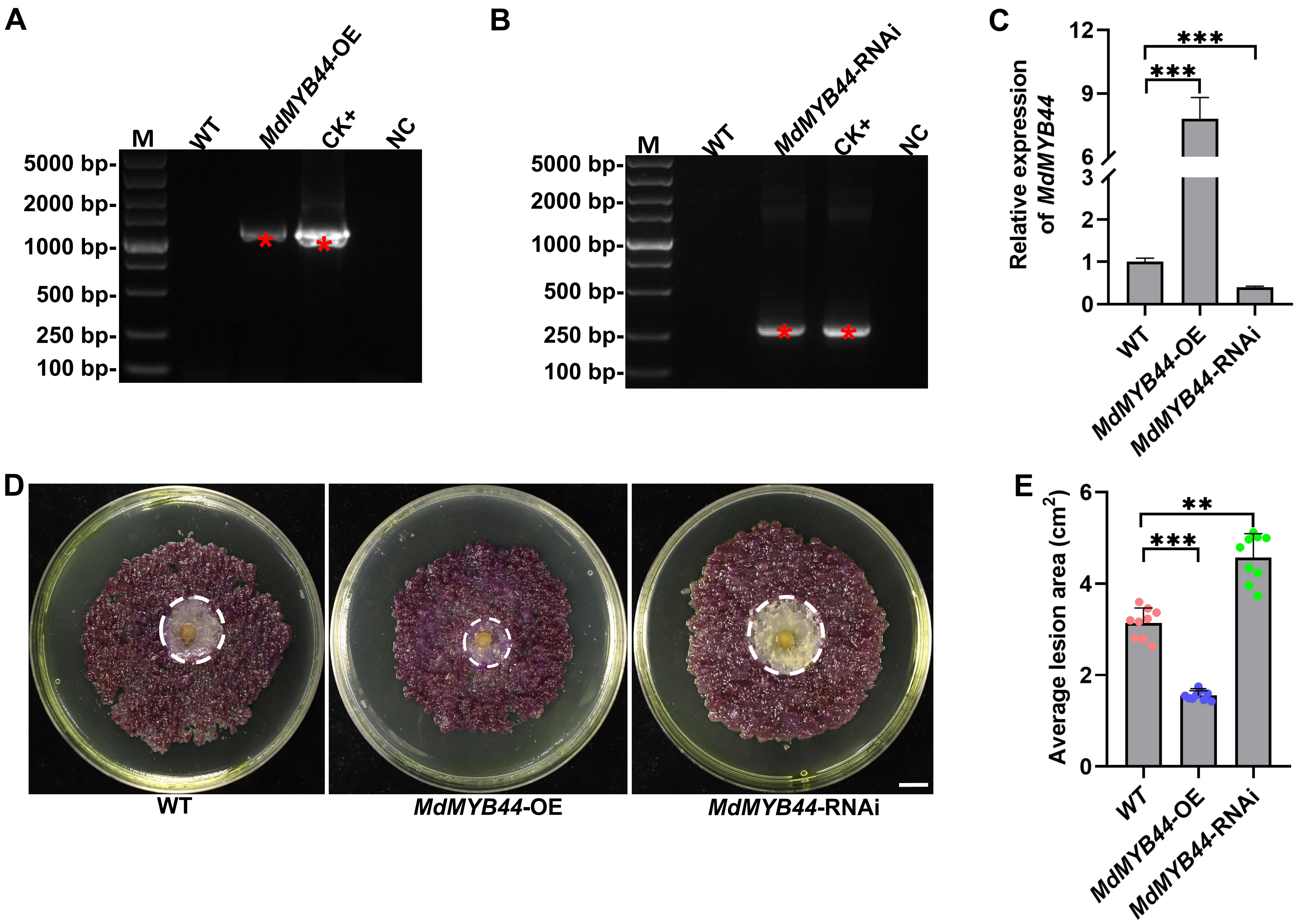

Supplemental Fig. S10

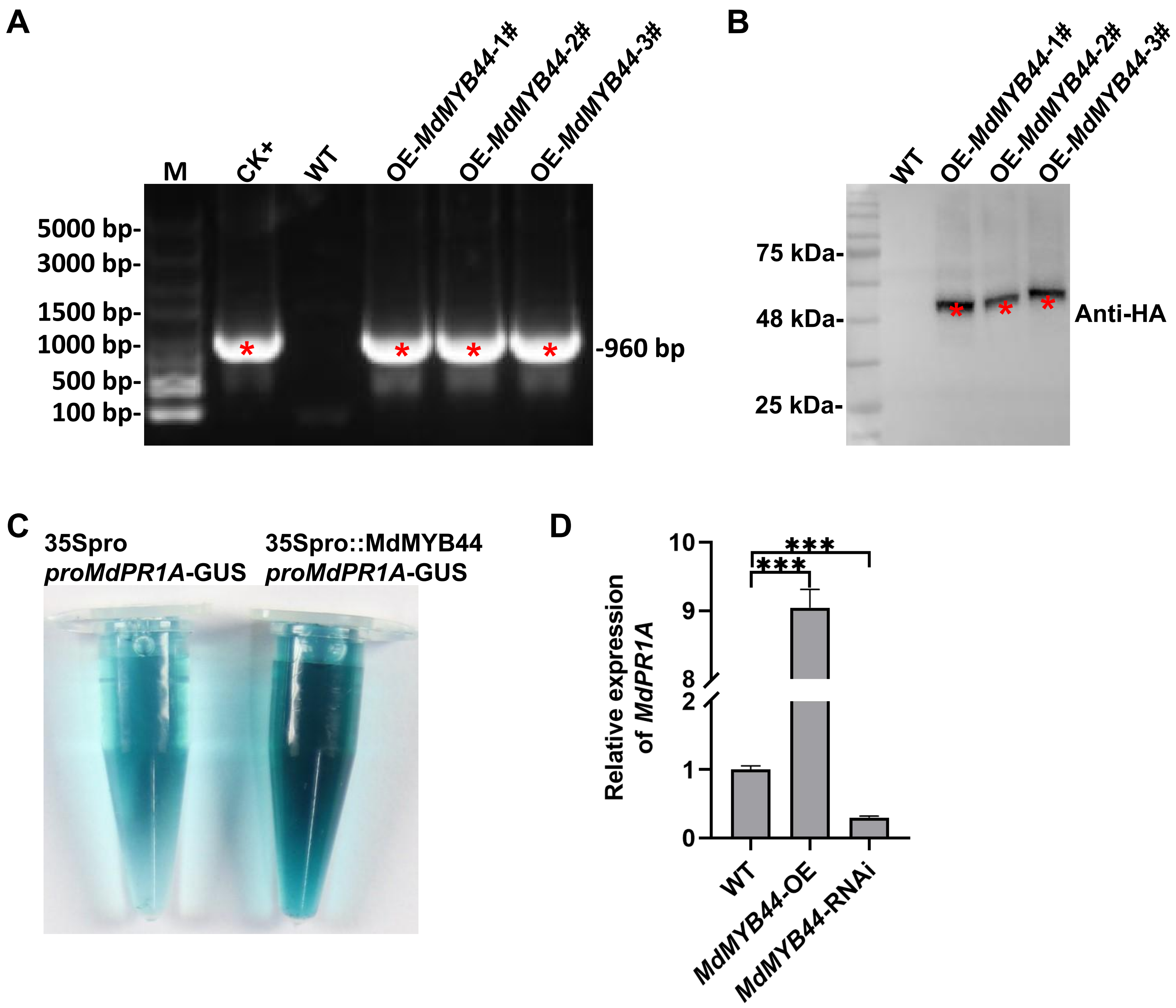

Supplement: Web_Material_uhag054 [file web_material_uhag054.zip › Supplemental FigureS 2026.1.29.pdf]
